# Inter-relationships between the economic and emotional consequences of colorectal cancer for patients and their families: a qualitative study

**DOI:** 10.1186/1471-230X-12-62

**Published:** 2012-06-07

**Authors:** Alan Ó Céilleachair, Liza Costello, Claire Finn, Aileen Timmons, Patricia Fitzpatrick, Kanika Kapur, Anthony Staines, Linda Sharp

**Affiliations:** 1 National Cancer Registry Ireland, Cork, Ireland; 2 National Economic and Social Council, Parnell Square, Dublin 1, Ireland; 3 School of Public Health, Physiotherapy & Population Science, University College Dublin, Dublin, Ireland; 4 School of Economics and Geary Institute, University College Dublin, Dublin, Ireland; 5 School of Nursing, Dublin City University, Dublin, Ireland; 6 National Cancer Registry, Building 6800, Cork Airport Business Park, Kinsale Road, Cork, Ireland

**Keywords:** Colorectal cancer, Costs & cost analysis, Patients, Caregivers, Social support, Employment

## Abstract

**Background:**

While the evidence-base concerning the economic impact of cancer for patients and their families/carers has grown in recent years, there is little known about how emotional responses to cancer influence this economic impact. We investigated the economic costs of cancer in the context of patients’ emotions and how these both shaped the patient and family burden.

**Methods:**

Health professionals from six hospitals invited patients diagnosed with colorectal cancer (ICD10 C18-C20) within the previous year to take part in the study. Semi-structured face-to-face interviews were conducted with patients and, where available, a family member. Interviews covered medical and non-medical costs incurred as a result of cancer and the impact of these on the lives of the patient and their family. Interviews were audio-recorded. Recordings were transcribed verbatim and these data were analysed qualitatively using thematic content analysis.

**Results:**

Twenty-two patients with colorectal cancer (17 colon and 5 rectal; 14 women and 8 men) were interviewed; 6 were accompanied by a family member. Important cancer-related financial outlays included: travel and parking associated with hospital appointments; costs of procedures; increased household bills; and new clothing. Cancer impacted on employed individuals’ ability to work and depressed their income. The opportunity cost of informal care for carers/family members, especially immediately post-diagnosis, was a strong theme. All patients spoke of the emotional burden of colorectal cancer and described how this burden could lead to further costs for themselves and their families by limiting work and hindering their ability to efficiently manage their expenses. Some patients also spoke of how economic and emotional burdens could interact with each other. Support from employers, family/carers and the state/health services and patients’ own attitudes influenced this inter-relationship.

**Conclusions:**

The economic impact of colorectal cancer on patients and their families is complex. This study suggests that the economic costs and the emotional impact of cancer are often related and can exacerbate each other, but that various factors can meditate this inter-relationship.

## Background

More than one million people are diagnosed with colorectal cancer (CRC) worldwide each year (Parkin, 2005). The disease is responsible for more than half a million deaths annually, mostly in developed countries [1,2]. Due to population ageing in developed countries, numbers of CRC cases are rising and are expected to continue rising in the coming years [2,3]. Survival too has been increasing [4]. As a result of these trends more people are living for longer with the disease.

This increase in new cases and survivors has implications for health services, patients and their families, and society as a whole. The National Cancer Institute estimated that the direct medical, indirect morbidity and indirect mortality costs of cancer in the United States in 2010 totalled over $260 billion [5]. CRC is thought to constitute more than 10% of this burden in the USA [6]. Emerging evidence from other settings also suggests the health services cost associated with CRC are significant [7-11]. As more people require treatment and treatments become more expensive, these costs are likely to increase. Evidence too has emerged, though in lesser quantities, on patient costs. One study estimated that time costs relating to treatment alone over a CRC patient's lifetime are in excess of $4,500 [12]. To date, studies of patient-borne costs have considered time and travel, out-of-pocket expenses (both medical and non-medical) and employment issues [12-14], although, in most instances, these have been assessed in isolation from each other, despite the fact that patients may experience costs concurrently. Furthermore, much of the previous research has been performed against the backdrop of either predominantly publicly-funded [13,15-17] or privately-funded healthcare systems [12,18].

Other costs of cancer for patients and their families include the emotional costs, which may be severe. Patients and caregivers may experience fear, anxiety, depression and anger [19-21]. While studies have examined the economic and emotional consequences of CRC, to the best of our knowledge there has been no investigation of the inter-relationships between the two. Given the complex nature of both of these burdens it is possible that there are important dynamics at work which might not be easily detected using quantitative research methods.

Using a qualitative approach, we aimed to investigate the broad spectrum of economic and emotional consequences faced by CRC patients, the inter-relationships between these and meditating factors. The setting was Ireland, which is one of the few countries where a universal access health system coexists with private health insurance (PHI) and where patients are free to move between the two. Moreover, the dispersed nature of the population [22] in conjunction with the ongoing centralisation of cancer services [23] could mean that patient issues, such as travel difficulties, emerge more strongly here than in other settings.

## Methods

### Setting

The public health system in Ireland is available to all but most pay for GP appointments, prescription drugs and make co-payments towards the cost of inpatient stays. Possession of a medical card covers these costs and co-payments. Medical cards are means-tested for people under 70 and, for most of the study period, were universally available to those aged 70 and older. Cancer patients without a medical card at the time of diagnosis may apply for a card afterwards on hardship grounds. The typical cost of a GP appointment is €50. The most commonly-held private PHI plans cost in the region of €1,000 per adult per annum and cover hospital in-patient stays, but not necessarily outpatient or primary care visits. The maximum co-payment a patient without either a medical card or PHI can make for an inpatient stay is €75 per day, up to a maximum of €750 in any 12 consecutive months. Public and private hospitals are often co-located and movement by patients between the two is common. Approximately half of the population is covered by PHI, 30% possess a medical card and 27% have neither [24].

### Subjects

The study was part of a larger programme of work investigating the overall economic impact of colorectal cancer. Subjects were recruited from six hospitals which included large Centres of Excellence [23], specialist oncology units and hospices. Patients were eligible if they had been diagnosed with a primary, invasive CRC (ICD 10: C18-C20) within the previous 12 months. Sampling was purposive to ensure diversity of age, gender, disease extent, treatment pathways, residence characteristics (urban or rural) and employment situations.

There was no relationship between the investigators and participants before the study began. Clinicians and specialist nurses involved in the care of CRC patients assisted with recruitment by making initial approaches to potentially eligible individuals. They explained the study and provided an information sheet which described the study aims in general terms (i.e. to investigate the economic impact of cancer on patients and their families). Details of those who were potentially interested in taking part were forwarded to the research team, one of whom contacted them by post and/or phone to confirm their willingness to be interviewed and arrange an interview date and location. Where appropriate, and with the agreement of the patient, the researcher also invited a family member (generally a spouse) involved in providing care or support to the patient (a carer) to be interviewed.

### Interviews

Interviews were conducted between August 2007 and October 2009. They were face-to-face, took place in a location of the participant’s choosing (usually the patient’s home), and lasted 60–90 minutes. Where present, carers were interviewed alongside the patient. Interviews were conducted by two team members (AOC, CF) who were already employed as researchers on the programme on the overall economic impact of cancer. They had both undergone training in qualitative methods and specifically in conducting interviews with cancer patients (Clinical Research Collaboration Cymru training programme). Before the interview, participants were given another chance to review the information sheet and ask any questions they might have. They then provided signed informed consent.

Interviews were semi-structured around a topic guide. This guide was informed by literature review, discussions with health professionals, a brainstorming session with a national bowel cancer support group and semi-structured interviews with hospital-based oncology social workers that were conducted for a parallel study involving patients with other cancers [25]. The topic guide covered: the socio-demographic characteristics of the patient and their family; the patients’ care pathway; the economic consequences of cancer for patients and their family/carers; and the emotional impact of cancer.

No repeat interviews were conducted. The interviewers liaised at several points during the course of the fieldwork to discuss data saturation. Recruitment ceased once new themes/issues stopped emerging.

### Analysis

With the participants’ consent, interviews were recorded and transcribed verbatim. Written notes of participants were also taken by the researchers during interviews. An experienced qualitative researcher (LC) undertook the analysis. This was a phenomenological study, in which emphasis was placed on the subjective meaning for individuals surrounding their experience [26], with the aim of exploring, in detail, how participants made sense of their personal and social world [27]. A thematic content analysis was conducted. All themes were derived from the data and not determined in advance. Interviews were coded and analysed manually, to facilitate an iterative approach, keeping track of individual experience, and how different factors and themes impact on this experience. Recordings and transcripts were listened to and read repeatedly. Codes and themes were identified through these repeat readings and review of the transcripts. Converging and diverging representations of each theme were identified across transcripts. A cross-comparative approach was taken, referring back and forth between the raw data, the codes and the emerging themes throughout the process of analysis, in order to confirm and validate conclusions drawn. Information on the impact of cancer on carers was provided by both the carers themselves and also by the patient where the carer was not present.

Transcripts were not returned to interviewees and they were not invited to provide feedback on the study’s findings, though they were offered a summary of the study results.

### Ethical approval

Approval for the study was obtained from the research ethics committees covering the six hospitals from which subjects were recruited, namely: Our Lady’s Hospice Harold’s Cross Research Ethics Committee; the Clinical Research Ethics Committee of the Cork Teaching Hospitals (for Cork University Hospital and Mercy University Hospital); St Luke's Hospital Research Management Committee; St Vincent’s Healthcare Group Ethics and Medical Research Committee; and Galway Research Ethics Committee (site specific approval for University College Hospital Galway).

## Results

### Characteristics of participants

In total, 22 patients and 6 carers (4 spouses and 2 daughters) were interviewed. Patients’ characteristics are shown in Table 1. They ranged in age from 44 to 82; 17 had been diagnosed with colon cancer and 5 with rectal cancer; 14 were women and 8 men; 13 lived in urban areas while 9 were from more rural parts of the country.

**Table 1:** Participant characteristics

***VARIABLE***	***GROUPING***	***N***
***Cancer site***	Colon:	17
	Rectal:	5
***Age***	less than 50 years:	2
	50-59 years:	2
	60-69 years:	12
	70 and older:	6
***Sex***	female:	14
	male:	8
***Area of residence***	urban^1^:	13
	rural:	9
***Medical card at time of interview***	yes:	16
	no:	6
***Private health insurance at diagnosis***	yes:	9
	no:	13^2^
***Employment status at diagnosis***	retired:	13
	working:	8
	unemployed:	1
***Received sick-pay from employer***	yes:	6
	no:	2
	not applicable:	14
***Marital status***	married:	13
	single:	3
	divorced:	1
	widow(er):	4
	cohabiting:	1

^1^ ‘Urban’ includes those residing in cities; ‘rural’ includes all other patients

^2^ One participant had neither health insurance nor a medical card.

### Themes

**Figure 1:**
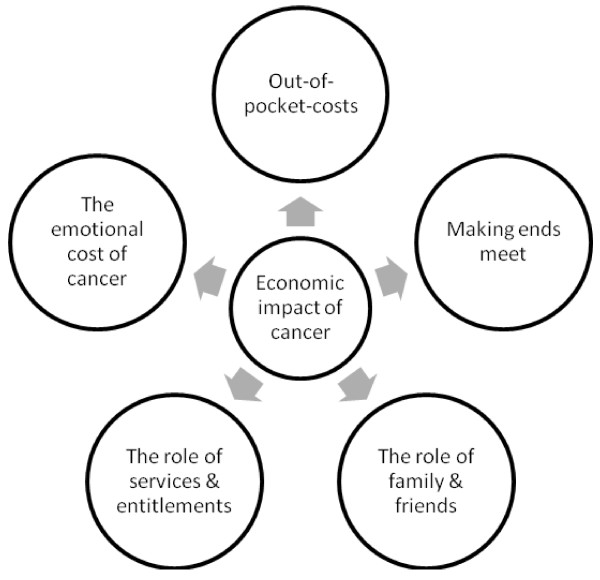
Themes arising from interviews with patients and carers.

The major themes that emerged from the interviews were: out-of-pocket costs (both medical and non-medical), “making ends meet” (i.e. managing financially), the role of family and friends, services and entitlements, and emotional costs (Figure 1).

### Out-of-pocket costs

Most respondents had a medical card, PHI, or (in some instances) both, and were largely satisfied with the cover these provided. None of those with a medical card reported any costs relating directly to their hospital care, GP visits or prescription items. However, some patients with PHI found themselves having to pay substantial amounts for outpatient appointments and for procedures/tests. In some cases the type and extent of these costs was an unwelcome surprise.

"That’s the number of visits we have – eight visits altogether. Eight visits to consultants and that would have come to a cost of €960 and the insurance paid €640 of that (Pt019)."

"I had probably three or four ultrasounds and scans. €200 a pop (Pt004)."

Those without medical cards noted that regular post-treatment GP visits constituted a substantial ongoing cost. Some also paid the full contribution of €85 per month to a publicly-subsidised prescription drug scheme when purchasing supportive medications. Several items had to be paid for in full by all respondents, most commonly over-the-counter painkillers and mouthwash for chemotherapy-induced ulcers. These were not regarded as significant expenses by patients.

Travel costs to and from the hospital for treatment were important for all interviewees, especially so for those who had chemotherapy and/or radiotherapy, because this required more hospital visits. Some spoke of the difficulties they experienced in using public transport, especially because of journey length or treatment side-effects.

"[It took] at least an hour, yeah. … It [the bus] leaves at ten to nine, oh more than an hour. It would be nearly two. Around 11 [o’clock] you get there, with all the stops and stuff (Pt020)."

Interviewees appreciated the efforts of some hospitals to provide free transport and/or parking. However the limitations of such schemes (scarcity in the case of parking and journey length in the case of free transport) sometimes rendered them functionally useless because patients were so frail.

"They’d provide me with transport if I wanted. … You could be two or three hours driving. And you wouldn’t be able for that, you know? (Pt037)."

Some respondents had increased utility bills following diagnosis, particularly for the telephone and heating. They noted that this was because they were at home more and because (neo)adjuvant therapies could make them more susceptible to feeling cold. For some, these extra costs caused real hardship.

"Our bills last year…were €370. He was so cold. … [It’s] gas and we would have … lighting as well. … And the heating on as well. …Oh God it [The cost] was dreadful. Really dreadful (Spouse of PT007)."

Other household expenses, such as home alterations required because of limitations in mobility following treatment (for example, one family had to build a downstairs extension as the patient could no longer manage the stairs), could be very high, but were incurred by few patients. Almost all patients described how treatment-related changes in their bodies, such as weight loss, and/or accommodating a stoma made it necessary for them to buy new clothes. The associated costs ranged from incidental to burdensome (when an entirely new wardrobe was required).

### Making ends meets

Eight interviewees were working at diagnosis. Six of these interviewees received some sick pay from their employers and all were very appreciative of employer understanding, both in terms of time off and sick pay provision. Despite this support, all employed participants, including those who received sick pay, noted a drop in income post-diagnosis; in some cases the drop was substantial. This was particularly so in situations where an individual had to retire early or work fewer hours.

"So I have stopped working in the afternoon. … I reckon I lost €4,000. You know eight months at about €500 (Pt015)."

Two respondents who were working at diagnosis retired immediately. Both were unhappy with having to leave work but felt compelled to do so.

"I worked in childcare. I was in the running of a pre-school. … I had to give it up. I didn’t have to but I felt, I was advised there was a risk of infection with small children. … And then – it’s a very physical job. They all need lifting (Pt025)."

Some carers also took time off work following the diagnosis. For some, this leave was unpaid or had to be taken as part of holiday entitlements. In one instance, where the patient was receiving palliative care, his daughter left her job to become his full-time carer.

A few interviewees were self-employed and a cancer diagnosis could be especially challenging for them. Generally, these individuals felt pressure to continue working regardless of their health, even though it might be difficult to cope. One entrepreneur described how his diagnosis nearly caused his business to fold.

"But …I had got behind…obviously sending out accounts….I was only half functioning and it took a while and that’s where I got in trouble you know (Pt004)."

Those in other areas of self-employment such as farming were able to draw on the support of family and friends in keeping their business going. One described how his son took annual leave to help on the farm following the initial diagnosis while ongoing support was provided by his wife and a neighbour.

Those who took time off work were anxious to return as soon as possible. Being on a reduced income was keenly felt. One interviewee sought to bring forward his surgery so that he could get back to work sooner.

"I said [to the consultant] ‘I’m not worried about the cancer … however, I do have a financial concern.’ Because he was saying ‘If I can’t get you an early December [surgery date] … we’ll leave it until January or maybe February.’ So more months waiting, more months on half pay. So he said ‘I’ll definitely get you in regardless, by early December’ (Pt012)."

For those not working at diagnosis, the financial and economic impact of cancer was even more pronounced; these individuals generally had smaller pre-diagnosis incomes and cancer-related costs could have a severe impact, reducing their means considerably. One patient described how the high cost of hospital parking meant that she could not afford a cup of tea in the canteen. Another noted:

"And then we’d have to be getting coffees and that up there [the hospital] sometimes and a sandwich, you know … So it cost a pretty packet (PT034)."

Some described having to cut back on spending to make ends meet. Some used their savings, and found that savings accumulated over years could be exhausted very quickly.

"Well I just had a bit of money put by. It wasn’t much… Take €50 from it. … Take [another] €50 and it would be gone then before you knew where you were (Pt007)."

Savings could also function as a buffer allowing families to maintain their standard of living in the face of increased expenditure and/or reduced income.

"I would've been in a bad way, I’d say, if I hadn’t have had them savings behind. We can live the lifestyle we’re used to because of those savings. … I mean they’re dwindling now but, thank God I had them (Pt034)."

### The role of family and friends

All patients received some practical support from families and/or friends. This took many forms including help at home or with travel; support in the form of regular visits while the patient was in hospital/convalescing; or financial assistance. Some patients came to rely on this support, especially with respect to travelling to and from hospital. Emotional support was also provided. Patients noted that a carer could sometime have a great impact on their wellbeing through the security provided by knowing that they (the carer) were there if needed. Patients were very appreciative of the practical and emotional support they received.

"They [ my sons and daughters] organised a kind of a roster. … So that there would be somebody in hand. As it happened when I got out after the blockage problem, my wife took sick herself. She got a blessed virus which really debilitated her and again the family had to organise and cook lunch or dinner for us, you know. (PT026)."

"my daughter is in Y. And she works up here in X. And she loops around to make sure [I’m ok]…if one of them isn’t checking on me the other one is. I am well minded (PT009)."

"I’d be lost without [my daughter]. (PT034)."

The role of family and friends in providing support was most pronounced for older respondents, especially where infirmity meant the patient’s spouse was unable to provide help. In contrast, there was also a strong sense among older patients that they needed to preserve their independence.

"I’d say “I’m fine” and then my neighbour next door, she would insist on driving me in [to the hospital]. It was driving me mad because I only had to go to the end of the hill to get the bus.... But I was fine and it’s nice to have a bit of independence. (Pt009)."

Patients were mindful that the support they received often protected them from a whole range of expenses that they would otherwise have had to bear. In some instances, they described how the support meant they could preserve their savings. All agreed that without this support their situation would have been much more challenging.

### Services & entitlements

In the main, patients were knowledgeable about the various potential supports that might be available to them within the health and social welfare system, such as the drug payment scheme or medical cards. This awareness came from a number of sources including health care providers and prior knowledge. Patients who were taking advantage of these services were quite happy with the cover provided. However, a number of difficulties were described. Some patients found the process of applying for a medical card unduly arduous and embarrassing. Such feelings were shared by those who had been successful in their application and those who had not.

"It [the medical card] was a bit hard to get. … Very, very hard. … They [the community welfare officers who adjudicate on applications] really humiliate you. It’s horrible (spouse of Pt007)."

Some poor experiences resulted from conflicting or confusing information about entitlements.

"I have a friend working in the Health Board ..... I remember at the time she had said to me that I should be entitled to a temporary medical card. . … When I went about it, I got a very negative kind of reaction. And I was told “no” (Pt016)."

Patients’ attitudes affected the way in which they engaged with services. One participant had, based on personal experiences, a negative opinion regarding benefits of any kind and did not seek support even where he knew it was available.

"You’ve only gurriers and gougers living around here. They’re drunk seven days a week and work is a dirty word. And yet I don’t know how I missed out on it [A perceived easy life on benefits]. St. Vincent de Paul is sending them to Wales for the summer. Not for two weeks, three weeks and where does PT007 go? (PT007)."

Others viewed benefits as entitlements following a lifetime of social insurance contributions and were comfortable accessing services or applying for benefits. One patient spoke of an active process of informing herself.

"I’m very good about whatever is there .......all the services are there and you would be a fool if you didn’t [take them up] (PT009)."

"The emotional impact of colorectal cancer"

All patients spoke about the emotional cost of CRC for them and their families. Even where economic issues were not a cause of worry in and of themselves, the emotional consequences took their toll.

"Financially, I had a good packet in the job, you know, so I’m not destitute by any stretch of the imagination. But I mean in terms of emotional stress and strains, stuff like that, this illness has cost us (PT034)."

Accessing health and social services could also cause emotional strain, because of unhelpful or insensitive staff, or due to the specific circumstances or requirements. For example, one patient was, unexpectedly, required to pay for scans before the results would be released. She described feeling threatened by this.

Some patients described how a lack of public knowledge or understanding of CRC could lead to social embarrassment. One patient described taking the bus home after chemotherapy:

"And I asked the bus driver would he close the door. Oh God. He turned around to the rest of the bus [and said] ‘This lady wants to close the door and we’re all dying with the heat, she says she’s cold***.’ …***Oh God. And I just wanted to explain ‘I’m not just cold, I’m sick.’ (PT025)."

For families too, the emotional impact of cancer could be significant. Often family members or carers had other stressors in their lives (such as pregnancy and moving house) and the cancer diagnosis represented an added emotional burden.

"It’s putting more pressure on them[family members or carers] and they are worried about you and everything else (Pt009)."

Interviewees described how the economic and emotional impacts of cancer were inter-related, both positively and negatively. One patient felt compelled to retire early from employment and was left with a deep sense of injustice about this. Another, who was successful in applying for a medical card, described the difference this made to her emotional wellbeing.

"I got ill just after I retired. … It wasn’t fair. Because all I done all my life was work, from the time I was eleven years old. In those days you didn’t pay tax or PRSI (Pt007)."

"That was such a blessing to get that medical card because it took [away] an awful lot of pressure and worry. (Pt025)."

In addition, the emotional strain could cause new economic concerns, on top of those resulting directly from the cancer diagnosis. In one instance a spouse who had already taken annual leave to be with her husband around the time of his diagnosis and treatment was forced to take more leave as a result of the emotional strain she had endured.

"She was quite upset. So she took that day completely off work. And there was scattered days she took off, you know (Pt012)."

For others, the stress that cancer caused meant they were less able to keep track of day-to-day spending.

"It could have been more [money spent] … See, I never kept notes. … See, I usually keep notes but I was so ill for him, that my mind… [was not as focused] (PT007)."

## Discussion

Our patients covered a range of ages, disease stages, care journeys, personal circumstances and socio-economic backgrounds. Colorectal cancer affects men and women in approximately equal measure; therefore we were able to ensure that gender perspectives on cancer and its economic and emotional impacts were considered. Our sample included several people whose cancer was no longer curable (, though none was at the very end of life). The issues faced by these patients were not fundamentally different to those of other patients who were cancer-free. The inclusion of carers and family members in our study added their perspective to that of patients and provided more insight into the experiences of patients themselves. Usually, carers’ perspectives are investigated separately from that of patients [12,15,16,18,19,28,29]. Markman et al (2010) [30] did explore cancer-related out-of-pocket costs from the perspective of the family as a whole but this study did not consider the multi-dimensional nature of the economic impact of the disease. The qualitative nature of our study allowed us to extend the existing body of evidence on “costs” of cancer (in its widest sense) and explore both economic and emotional costs, how these interact, and how patient and family experiences, attitudes, and external supports influence them. These issues would be unlikely to emerge from a quantitative study.

In recent years, a consensus has begun to emerge regarding the core components of the economic burden of cancer for patients and their families [13,30,31]. Cancer-related expenses may include treatment costs (particularly in private healthcare systems), treatment-related travel and parking costs, household costs, and personal care costs (such as clothing). However, much of the previous literature pertains to breast and prostate cancer; CRC has been relatively under investigated. The age and socio-economic distribution of CRC differs from breast and prostate cancer and treatment patterns differ [32], making it possible that the nature of costs and their impact on patients will differ. In fact, our findings were largely consistent with the literature, suggesting that, at least in part, the mere fact of having a cancer is what impacts on patients economically, irrespective of the type of cancer. Furthermore, it is worth noting that private health insurance did not provide any protection from non-medical costs; all patients we interviewed bore such costs.

**Figure 2:**
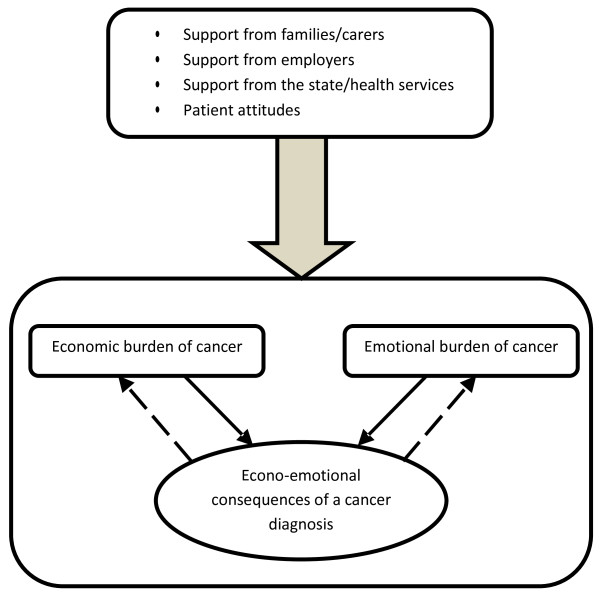
Conceptual model of interrelationship between economic and emotional consequences and mediating factors.

Although economic costs are important in and of themselves, the interplay between these costs and the emotional toll of CRC upon patients and their families is of equal importance. It is clear from this study and others that cancer causes emotional strain for patients and their families [31,33]. Moreover, some have shown that financial distress in cancer patients can also cause emotional difficulties [34,35]. Our study suggests that this is a two-way relationship (see Figure 2). Not only can economic strain cause emotional distress, the emotional toll of colorectal cancer has the potential to influence the economic situation of the patient and caregiver beyond the effect of the diagnosis itself. This gives rise to a situation where a family could face a direct economic loss from cancer that was exacerbated by further economic losses relating to their emotional state following diagnosis. This in turn could cause yet more emotional problems. This negative feedback between economic and emotional consequences of cancer has the potential to make difficult situations even worse.

Our findings further suggest that the factors that can exacerbate, alleviate, moderate or prevent this negative feedback loop are multi-faceted. The patient’s employment circumstances and income are of major importance. Our results would suggest that patients experience these issues not just in directly monetary terms but in relation to how they impact on their own lives and those of their families.

Sick pay has previously been shown to be an important determinant of work resumption post-cancer in Ireland [36]. This study reveals that it is an important mediator of the economic-emotional impact of the disease. With adequate sick pay, the loss of income was greatly reduced and this in turn meant that patients worried less about their financial situation; hence financial stress was not added to the stress due to the cancer diagnosis and could not exacerbate any existing economic issues for the patient. On the other hand, in some instances, the consequences of being absent from work with inadequate sick pay were such that they influenced treatment decisions. This finding is consistent with the existing literature [37,38].

The availability of familial support can also impact on the emotional-economic strain for patients [39]. Using qualitative methods, Bradley et al [31] examined the role of family and friends in providing economic support, but did not consider the wider implications of this support on patient wellbeing. In our study, patients frequently spoke at great length about the things that family and friends had done to improve their lives, or to show that they cared, or to help out financially. This meant that, through the attention of family/friends, patients often avoided a great degree of economic outlay and emotional strain. The importance of support provided by family in this study is consistent with findings of another Irish study of breast, prostate and lung cancer patients [25] and may reflect strong family cohesion in Ireland [40] compared to other countries in northern Europe. Further research would be valuable in clarifying whether the role of family support is as strong in other settings.

Patients themselves could influence the cycle of economic and emotional strain by engaging with available health and social welfare services. A small number of patients had received conflicting or wrong information on their entitlement to supports, especially medical cards. This often led to confusion and embarrassment for patients when they were informed that their application for support had failed. Evidence from the UK suggests that benefits available to cancer patients often go unclaimed [34], meaning that patients and their families are assuming unnecessary burdens. This observation, together with our findings, suggests that more and better communication with cancer patients regarding their entitlements is needed. There is also a need to provide this information in a way that avoids triggering issues around benefits that can prevent people from claiming these even when entitled (such as stigma) [41].

## Conclusions

Our study shows that the economic impact of colorectal cancer for patients and their families is complex and suggest that there is a strong inter-relationship between the economic and emotional impact of the disease. Support from the family, workplace and state/health services, and patients’ own attitudes can interrupt this negative cycle, allowing patients and families to cope better with what is already an incredibly challenging time in their lives. Further studies are needed to confirm these findings in other health and social welfare settings.

## Abbreviations

CRC: Colorectal cancer; PHI: Private health insurance.

## Competing interests

The authors have no competing interests.

## Authors’ contributions

AÓC developed the topic guide, liaised with clinical and nursing staff, arranged and conducted the patient interviews. He contributed to the analysis of the interview transcripts and drafted the paper.CF liaised with clinical and nursing staff, arranged and conducted patient interviews and commented critically on the paper. LC contributed to the analysis and interpretation of the interview transcripts and commented critically on the paper. AT contributed to design and implementation of the study methods, development of the topic guide, and commented critically on the paper. PF contributed to design and implementation of the study methods and commented critically on paper. KK contributed to design and implementation of the study methods and commented critically on the paper. AS contributed to design and implementation of the study and commented critically on the paper. LS contributed to design and implementation of the study, the development of the topic guide, analysis and interpretation of the interview transcripts, and assisted with writing the paper. All authors read and approved the final manuscript.

## Pre-publication history

The pre-publication history for this paper can be accessed here:

http://www.biomedcentral.com/1471-230X/12/62/prepub

## References

[B1] American Cancer Society Global Cancer Facts & Figures 2007 American Cancer Society, Atlanta

[B2] Verdecchia A Francisci S Brenner H Gatta G Micheli A Mangone L Kunkler I EUROCARE-4 Working Group Recent cancer survival in Europe: a 2000–02 period analysis of EUROCARE-4 data Lancet Oncol 2007 8 9 784 796 10.1016/S1470-2045(07)70246-2 17714993

[B3] Comber H Walsh P Patterns Of Care And Survival Of Cancer Patients In Ireland 1994 To 2004 2008 National Cancer Registry, Cork

[B4] Verdecchia A Santaquilani M Sant M Survival for cancer patients in Europe Ann Ist Super Sanita 2009 45 3 315 324 19861737

[B5] American Cancer Society Cancer Facts & Figures 2007 American Cancer Society, Atlanta

[B6] Mariotto AB Yabroff KR Shao Y Feuer EJ Brown ML Projections of the cost of cancer care in the United States: 2010–2020 J Natl Cancer Inst 2011 103 2 117 128 10.1093/jnci/djq495 21228314 PMC3107566

[B7] Bending MW Trueman P Lowson KV Pilgrim H Tappenden P Chilcott J Tappenden J Aalto P Estimating the direct costs of bowel cancer services provided by the National Health Service in England Int J Technol Assess Health Care 2010 26 4 362 369 10.1017/S0266462310001078 20942988

[B8] Mcafee DA West J Scholefield JH Whynes DK Hospital costs of colorectal cancer care Clin Med Oncol 2009 20 3 27 37 10.4137/cmo.s2362 PMC2872591 20689608

[B9] Yabroff KR Warren JL Schrag D Mariotto A Meekins A Topor M Brown ML Comparison of approaches for estimating incidence costs of care for colorectal cancer patients Med Care 2009 47 7 Suppl 1 S56 S63 19536010 10.1097/MLR.0b013e3181a4f482

[B10] Clerc L Jooste V Lejeune C Schmitt B Arveux P Quantin C Faivre J Bouvier AM Cost of care of colorectal cancers according to health care patterns and stage at diagnosis in France Eur J Health Econ 2008 9 4 361 367 10.1007/s10198-007-0083-0 18030510

[B11] Paramore L Thomas S Knopf KB Cragin LS Fraeman KH Estimating costs of care for patients with newly diagnosed metastatic colorectal cancer Clin Colorectal Cancer 2006 6 1 52 58 10.3816/CCC.2006.n.021 16796792

[B12] Yabroff KR Warren JL Knopf K Davis WW Brown ML Estimating patient time costs associated with colorectal cancer care Med Care 2005 43 7 640 648 10.1097/01.mlr.0000167177.45020.4a 15970778

[B13] Longo CJ Deber R Fitch M Williams AP D'Souza D An examination of cancer patients' monthly 'out-of-pocket' costs in Ontaria, Canada Eur J Cancer Care 2007 16 6 500 507 10.1111/j.1365-2354.2007.00783.x 17944764

[B14] Sanchez KM Richardson JL Mason HR The return to work experiences of colorectal cancer survivors AAOHN J 2004 52 12 500 510 15635931

[B15] Longo CJ Bereza BG A comparative analysis of monthly out-of-pocket costs for patients with breast cancer as compared with other common cancers in Ontario, Canada Curr Oncol 2011 18 1 e1 e8 21331267 10.3747/co.v18i1.681 PMC3031360

[B16] Longo CJ Fitch M Deber RB Williams AP Financial and family burden associated with cancer treatment in Ontario, Canada Support Care Cancer 2006 14 11 1077 1085 10.1007/s00520-006-0088-8 16896878

[B17] Syse A Tretli S Kravdal O The impact of cancer on spouses' labor earnings: a population-based study Cancer 2009 115 18 Suppl 4350 4361 19731350 10.1002/cncr.24582

[B18] Yabroff KR Kim Y Time costs associated with informal caregiving for cancer survivors Cancer 2009 115 18 Suppl 4362 4373 19731345 10.1002/cncr.24588

[B19] Worster B Holmes S A phenomenological study of the postoperative experiences of patients undergoing surgery for colorectal cancer Eur J Oncol Nurs 2009 13 5 315 322 10.1016/j.ejon.2009.04.008 19482512

[B20] Julkunen J Gustavsson-Lilius M Hietanen P Anger expression, partner support, and quality of life in cancer patients J Psychosom Res 2009 66 3 235 244 10.1016/j.jpsychores.2008.09.011 19232236

[B21] Cotrim H Pereira G Impact of colorectal cancer on patient and family: implications for care Eur J Oncol Nurs 2008 12 3 217 226 10.1016/j.ejon.2007.11.005 18567538

[B22] Eurostat Eurostat regional yearbook 2010 2010 Publications Office of the European Union, Luxembourg

[B23] The National Cancer Forum A Strategy for Cancer Control In Ireland 2006 Department of Health & Children, Dublin

[B24] Central Statistics Office Health Status & Health Service Utilisation 2008 The Stationery Office, Cork

[B25] Sharp L Timmons A The Financial Impact of a Cancer Diagnosis 2010 National Cancer Registry, Cork

[B26] Creswell JW Maietta RC Miller DC, Salkind NJ Qualitative Research Handbook of social research 2008 Sage, Thousand Oaks, California

[B27] Smith JA Osborn M Smith JA Interpretative Phenomenological Analysis Qualitative psychology: A practical guide to methods 2008 Sage, London

[B28] Van Houtven CH Ramsey SD Hornbrook MC Atienza AA van Ryn M Economic burden for informal caregivers of lung and colorectal cancer patients Oncologist 2010 15 8 883 893 10.1634/theoncologist.2010-0005 20667966 PMC3228017

[B29] Yabroff KR Davis WW Lamont EB Fahey A Topor M Brown ML Warren JL Patient time costs associated with cancer care J Natl Cancer Inst 2007 99 1 14 23 10.1093/jnci/djk001 17202109

[B30] Markman M Luce R Impact of the cost of cancer treatment: an internet-based survey J Oncol Pract 2010 6 2 69 73 10.1200/JOP.091074 20592778 PMC2835484

[B31] Bradley S Sherwood PR Donovan HS Hamilton R Rosenzweig M Hricik A Newberry A Bender C I could lose everything: understanding the cost of a brain tumor J Neurooncol 2007 85 3 329 338 10.1007/s11060-007-9425-0 17581698

[B32] Faggiano F Partanen T Kogevinas M Bofetta P Socioeconomic differences in cancer incidence and mortality Social Inequalities and Cancer. No. 138 edition 1997 65 176 9353664

[B33] Wagner EH Aiello Bowles EJ Greene SM Tuzzio L Wiese CJ Kirlin B Clauser SB The quality of cancer patient experience: perspectives of patients, family members, providers and experts Qual Saf Health Care 2010 19 6 484 489 10.1136/qshc.2010.042374 21127109 PMC13007532

[B34] Moffatt S Noble E Exley C "Done more for me in a fortnight than anybody done in all me life." How welfare rights advice can help people with cancer BMC Health Serv Res 2010 3 10 10.1186/1472-6963-10-259 PMC2941682 20815908

[B35] Sharp L Carsin AE Timmons A Associations between cancer-related financial stress and strain and psychological wellbeing among individuals living with cancer Psychooncologyin press 10.1002/pon.3055 22411485

[B36] Sharp L Timmons A Social welfare and legal constraints associated with work among breast and prostate cancer survivors: Experiences from Ireland J Cancer Survivin press 10.1007/s11764-011-0183-9 21681406

[B37] Hofstatter EW Understanding patient perspectives on communication about the cost of cancer care: a review of the literature J Oncol Pract 2010 6 4 188 192 10.1200/JOP.777002 21037869 PMC2900868

[B38] McFarlane J Riggins J Smith TJ SPIKE$: a six-step protocol for delivering bad news about the cost of medical care J Clin Oncol 2008 26 25 4200 4204 10.1200/JCO.2007.15.6208 18757335

[B39] Illingworth N Forbat L Hubbard G Kearney N The importance of relationships in the experience of cancer: a re-working of the policy ideal of the whole-systems approach Eur J Oncol Nurs 2010 14 1 23 28 10.1016/j.ejon.2009.06.006 19748315

[B40] Reher DS Family ties in Western Europe: persistent contrasts Popul Dev Rev 1998 24 2 203 234 10.2307/2807972

[B41] Chapple A Ziebland S McPherson A Summerton N Lung cancer patients' perceptions of access to financial benefits: a qualitative study Br J Gen Pract 2004 54 505 589 594 15296557 PMC1324838

